# Functional ability in younger and older elderlies after discharge from the intensive care unit. A prospective cohort

**DOI:** 10.5935/0103-507X.20170055

**Published:** 2017

**Authors:** Camila Dietrich, Juliana Rezende Cardoso, Fernanda Vargas, Evelin Carneiro Sanchez, Francine Hoffmann Dutra, Cátia Moreira, Marina Bessel, Caroline Robinson, Maicon Falavigna, Cassiano Teixeira

**Affiliations:** 1 Postgraduate Program in Rehabilitation Sciences, Universidade Federal de Ciências da Saúde de Porto Alegre - Porto Alegre (RS), Brazil.; 2 Hospital Moinhos de Vento - Porto Alegre (RS), Brazil.

**Keywords:** Critical care, Physical fitness, Frail elderly, Aging, Aged, Aged, 80 and over, Quality of live

## Abstract

**Objective:**

To compare the functional capacity of younger elderly individuals (60 to 79 years old) with that of older elderly individuals (≥ 80 years old) during the first 6 months after discharge from the intensive care unit.

**Methods:**

A multicenter prospective cohort study was conducted, in which data on intensive care unit admission and outcomes after hospital discharge (immediate post-discharge, after 3 months and after 6 months) were collected. Muscle strength was evaluated through the protocol of the Medical Research Council and dynamometry (handgrip); the ability to perform activities of daily life and functional independence were assessed by the Barthel index and the usual level of physical activity (International Physical Activity Questionnaire); and quality of life was assessed by the 12-Item Short-Form Health Survey Version 2.

**Results:**

Among the 253 patients included, 167 were younger elderly (between 61 and 79 years old), and 86 were older elderly (≥ 80 years old). During the sixth month of evaluation, the older elderlies presented a higher need for a caregiver (69.0% *versus* 49, 5%, p = 0.002). Functional capacity prior to intensive care unit admission and in the third month after discharge was lower in older elderlies than in younger ones (Barthel prior to the intensive care unit: 73.0 ± 30.0 *versus* 86.5 ± 22.6; p <0.001, Barthel in the third month: 63.5 ± 34.0 *versus* 71.5 ± 35.5, p = 0.03), as was the usual level of physical activity (International Physical Activity Questionnaire in the third month: active/very active 3.4% *versus* 18.3%, no physical activity 64.4% *versus* 39.7%, p < 0.001, and International Physical Activity Questionnaire in the sixth month: active/very active 5.8% *versus* 20.8%, no physical activity 69.2% *versus* 43.4%, p = 0.005). Older elderlies had lower muscle strength when assessed according to handgrip in both the dominant (14.5 ± 7.7 *versus* 19.9 ± 9.6, p = 0.008) and non-dominant limb (13.1 ± 6.7 *versus* 17.5 ± 9.1, p = 0.02). There were no differences in functional capacity loss or reported quality of life between the age groups.

**Conclusion:**

Although there were great functional capacity losses after discharge from the intensive care unit in both age groups, there was no difference in the magnitude of functional capacity loss between younger (60 to 79 years) and older elderly individuals (≥ 80 years old) during the first 6 months after discharge from the intensive care unit.

## Abstract (pt)

**Objetivo:**

Comparar a capacidade funcional de indivíduos idosos (60 a 79 anos) com a dos idosos mais velhos (≥ 80 anos) nos primeiros 6 meses após a alta da unidade de terapia intensiva.

**Métodos:**

Coorte prospectiva multicêntrica, na qual foram coletados dados referentes à internação na unidade de terapia intensiva e aos desfechos após a alta hospitalar (no pós-alta imediato, após 3 meses e após 6 meses). A força muscular foi avaliada por meio do protocolo do *Medical Research Council* e da dinamometria (preensão palmar); a capacidade de execução das Atividades de Vida Diária e independência funcional pelo índice de Barthel e pelo nível habitual de atividade física (*International Physical Activity Questionnaire*); e a qualidade de vida pelo *12-Item Short-Form Health Survey* Versão 2.

**Resultados:**

Dentre os 253 pacientes incluídos, 167 eram idosos entre 61 a 79 anos, e 86 eram idosos mais velhos. Os idosos mais velhos, no sexto mês de avaliação, apresentaram maior necessidade de cuidador (69,0% *versus* 49,5%; p = 0,002). A funcionalidade prévia à unidade de terapia intensiva e no terceiro mês após alta foi menor nos idosos mais velhos em comparação aos mais jovens (Barthel anterior à unidade de terapia intensiva: 73,0 ± 30,0 *versus* 86,5 ± 22,6; p < 0,001; Barthel no terceiro mês: 63,5 ± 34,0 *versus* 71,5 ± 35,5; p = 0,03), assim como o nível habitual de atividade física (*International Physical Activity Questionnaire* no terceiro mês: ativo/muito ativo 3,4% *versus* 18,3%; nenhuma atividade física 64,4% *versus* 39,7%; p < 0,001; e *International Physical Activity Questionnaire* no sexto mês: ativo/muito ativo 5,8% *versus* 20,8%; nenhuma atividade física 69,2% *versus* 43,4%; p = 0,005). Os idosos mais velhos apresentaram menor força muscular ao serem avaliados pela preensão palmar no membro dominante (14,5 ± 7,7 *versus* 19,9 ± 9,6; p = 0,008) e do não dominante (13,1 ± 6,7 *versus* 17,5 ± 9,1; p = 0,02). Não houve diferença na perda da funcionalidade e na qualidade de vida referida, entre os grupos etários.

**Conclusão:**

Mesmo com grande perda funcional após a alta da unidade de terapia intensiva em ambos os grupos etários, não houve diferença na magnitude da perda da funcionalidade de indivíduos idosos (60 a 79 anos) quando comparados aos idosos mais velhos (≥ 80 anos) nos primeiros 6 meses após a alta da unidade de terapia intensiva.

## INTRODUCTION

With the aging of the population, the number of elderly people admitted to intensive care units (ICUs) has increased. More than half of ICU admissions are related to individuals aged 65 and over.^(1)^ There are many factors that make elderly patients vulnerable to acute life-threatening events and the consequent need for intensive care: reduced physiological reserve, immunosenescence, presence of comorbidities, institutionalization, frequent hospitalizations and reduced access to health care. However, intensive medicine has allowed a growing number of patients to survive what used to be fatal illnesses.^(2)^

Due to this growing increase in the number of elderly people who need to be admitted to the ICU, we elaborate our work focused on this population, which was divided between two age groups: younger elderlies (60 to 79 years of age) and older elderlies (80 years of age or older). Many studies with elderlies show worsening functionality and quality of life after admission to the ICU but do not show any differences between the elderly and older elderly populations. This study considered clinical outcomes and interventions, taking relevant data that denote the need for investment during ICU admission, regarding the patient's age, based on post-discharge and long-term outcomes.

The objective of this study was to compare the functional capacity of younger elderlies (60 to 79 years old) with that of older elderlies (≥ 80 years old) during the first 6 months after discharge from the ICU.

## METHODS

This multicenter prospective cohort study was conducted in two hospitals in the Southern Region of Brazil - *Hospital Moinhos de Vento* and *Hospital Irmandade Santa Casa de Misericórdia* in Porto Alegre from May 2014 to December 2015. Patients older than 60 years who were in the period of 24 to 120 hours of discharge from the ICU were eligible. Exclusion criteria were: ICU stay for less than 72 hours when the reason for admission was clinical urgency or surgical urgency; elective surgery with recovery under the ICU protocol, whose length of stay in the unit was less than 120 hours; admission to the ICU by direct transfer from the ICU of another hospital; patients in respiratory isolation after discharge from the ICU; discharge or hospital transfer from the ICU; lack of telephone contacts; and the inability to sign the Informed Consent Form. Among the participants of this study, we selected elderly patients (≥ 60 years) for the present study.

Data collection from the baseline was performed between 24 and 120 hours after discharge from the ICU while the patient was still hospitalized (immediate discharge from the ICU or baseline). The patient was invited to participate in the study, and the acceptance was given by completion of the Informed Consent Form. In cases where the patient did not have physical or cognitive condition for consent, the same was obtained from a first-degree relative who was responsible for the patient. An interview with sociodemographic questions was conducted, and information on health and life habits related to the 3 months prior to admission was obtained from the patient or his relative. Next, the evaluation of the degree of functional dependence related to the 3 months prior to admission was performed using the Barthel index,^(3)^ which was answered by the family member when necessary. The Medical Research Council (MRC) peripheral muscle strength protocol was applied^(4)^ to evaluate the muscle strength of the lower and upper limbs, and handgrip strength was evaluated by manual dynamometry.^(5)^ Patients in contact isolation did not perform dynamometry due to the complexity of the equipment asepsis.

The data referring to ICU admission were collected retrospectively from the patient's chart, namely, reason for admission, severity scores, comorbidities, need for life support (ventilatory support, hemodynamics, dialysis, among others), length of hospitalization, complications and intercurrences during hospitalization.

Telephone follow-up interviews occurred 3 and 6 months after discharge from the ICU and were performed from the telephone center located at Moinhos de Vento Hospital. A patient was considered lost to follow-up when the telephone line provided by the patient was deactivated or non-existent or after ten failed contact attempts on different days and at different times within 25 days before and after the estimated date for the follow-up. The estimated follow-up date was calculated based on the ICU discharge date. A trained researcher conducted all of the interviews, following a structured script that contained the interviewer's presentation and the collection instruments. All interviews were recorded with the consent of the interviewees.

In the telephone follow-up at 3 months after discharge from the ICU, the subjects were asked about occurrences of readmissions and maintenance of specialized follow-ups (physiotherapy, speech therapy, among others); the Barthel index was also applied. This information was obtained from the family member responsible for the patient whenever necessary. The 12-Item Short-Form Health Survey Version 2 (SF12v2)^(6)^ and the short version of the International Physical Activity Questionnaire (IPAQ)^(7)^ were applied to evaluate the patient's health-related quality of life and their level of physical activity, respectively. These two instruments were applied only to the patient if he presented the physical and cognitive capacity to answer them. At 6 months after discharge from the ICU, telephone follow-up was repeated, and the same questions from the 3-month follow-up period were asked.

The Barthel index^(3)^ belongs to the Activities of Daily Living (ADL) evaluation field and measures functional independence regarding personal care and mobility. Scoring ranges from zero to 100 in 5-point intervals; higher scores indicate greater independence.^(3)^ The degree of dependence was established in five categories, according to the total score reached: total dependence (zero - 24), almost total dependence (25 - 50), moderate dependence (51 - 75), little dependence (76 - 99) and independence (100).^(8)^

The MRC^(4)^ is an instrument that evaluates the force of muscle contraction against the resistance of either gravity or the evaluator. Its ordinal score ranges from zero (no contraction) to 5 (normal muscle strength) for each of the 12 muscle groups. Thus, the total score ranges from zero to 60. The total value ≤ 48 is considered a cutoff point for muscle weakness.^(4)^ Patients unable to move at least one limb, regardless of cause, did not perform this evaluation.

Manual dynamometry was performed using a Saehan dynamometer and following the protocol suggested by the American Association for Hand Surgery^(5)^ to evaluate the handgrip strength, providing an estimate of the isometric strength at the upper end. The results are effectively correlated with strength in other muscle groups and are considered a good indicator of total muscle strength.^(5)^ Patients in contact isolation and those who could not be adequately positioned did not perform dynamometry. The SF12v2 instrument^(6)^ is a widely used scale in the assessment of health-related quality of life, resulting in scores ranging from zero to 100, with higher numbers indicating a better perception of quality of life. The instrument makes possible the separate evaluation for the mental and physical components of quality of life. The purpose of the IPAQ is to estimate the habitual level of physical activity^(7)^ by allowing its classification into levels of intensity. This instrument generates information regarding the frequency and duration of activities performed within the last 7 days.^(9)^

Because this study involved the subanalysis of a prospective cohort, the eligibility criteria were not specifically designed for this study. Although all patients admitted to the ICU were screened for the cohort upon discharge, some eligibility criteria could bias the result, such as the impossibility of performing the baseline interview with patients in respiratory isolation, patients transferred to another hospital or those who had been discharged from the ICU directly to their homes. Patients admitted to the ICU for elective surgery had entry criteria different from the criteria for those admitted for clinical complications or emergency surgery due to the design of the follow-up of the prospective cohort. Data regarding ICU admission, such as age, comorbidities, interventions and outcome, were taken from the patient's electronic records, avoiding memory bias. Although the study personnel they were not the same evaluators who performed the data collection, they were all trained for the process and were given initial instructions, followed up with collections and were monitored during the first quality control interviews, reducing calibration bias. The possibility of memory bias inherent to studies with retrospective information was reduced because at no time did the participants compare previous situations with the current one. The participants were always asked about the previous situation (3 months before admission at discharge from the ICU) and the current situation (after discharge and at 3 and 6 months).

### Statistical analysis

Categorical variables are described as absolute and relative frequencies (percentage), and continuous variables are described as averages and standard deviations. The comparison between the two age groups was performed by the chi-square test for dichotomous variables and by analysis of variance (ANOVA) for continuous variables. For variables that did not follow a normal distribution, the Kruskal-Wallis test was used. To estimate the association between outcome and predictor, Poisson regression was performed with robust variance or multinomial logistic regression, depending on the number of categories. For continuous outcomes, the association was analyzed using multiple linear regression. The regression model was adjusted for the Charlson comorbidity index, the Acute Physiology and Chronic Health Evaluation II (APACHE II) and the admission regime (health insurance/Unified Health System [Sistema Único de Saúde - SUS]). The level of significance was 5%. Analyses were performed using Statistical Analysis Software (SAS) version 9.4.

### Ethical approval

This study was nested to the prospective and multicentric cohort of Quality of Life after ICU Discharge, approved by the Research Ethics Committees of the participating institutions under opinion 935.342 and is in accordance with Resolution 466/12 of the National Health Council and the Declaration of Helsinki. All procedures involving participants were performed only after they signed the Informed Consent Form.

## RESULTS

**Figure 1:**
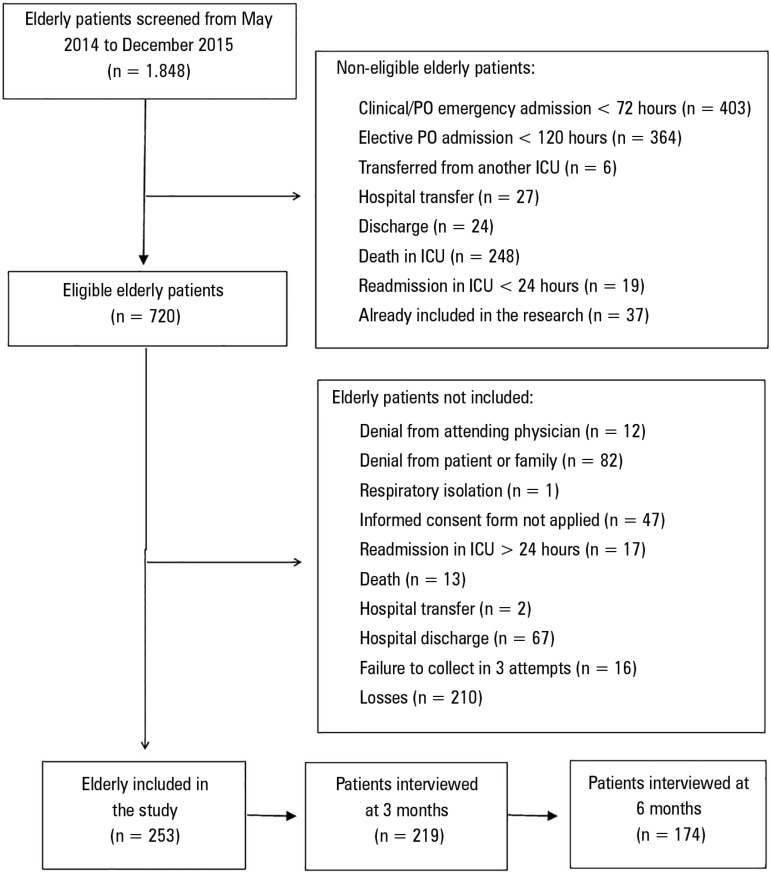
Elderly patients recruited, eligible and included during the baseline, and follow-up at 3 and 6 months after discharge from the intensive care unit. PO - postoperative; ICU - intensive care unit.

The multicenter study tracked 3243 discharges in both ICUs over a 19-month period. Of these, 1,848 were elderly patients, and 720 were eligible patients. The reasons for ineligibility and non-inclusion were described in the flowchart (Figure 1). The 253 elderlies included in the study were discharged from the ICU and then divided into two groups: younger elderlies between 61 and 79 years old (n = 167) and older elderlies ≥80 years old (n = 86).

After ICU discharge, still during the hospital admission period, there were 34 deaths. In the 3-month telephone follow-up interview, 45 patients died, and 219 were interviewed. In the follow-up of 6 months, 6 patients died, and 174 were interviewed. During the 6-month period, 9.8% of the sample was lost to follow up.

The sociodemographic data, reasons for ICU admission, comorbidities and functional status prior to ICU stay are shown in table 1. Table 2 shows the interventions performed during ICU stay and the outcomes during hospitalization and during the first week after discharge from the ICU.

**Table 1:** Characterization of the sample

Variables	Between 61 and 79 years old (N = 167)	≥ 80 years old (N = 86)	p value
Sociodemographic			
Male	87 (52.1)	43 (50.0)	0.75
Schooling (years)	9.2 ± 5.2	8.9 ± 5.58	0.68
Income per capita (BRL)	2.685 ± 3.430	4.442 ± 4.886	0.008
Health expenditures (BRL)	1.268 ± 2.405	1.817 ± 2.246	0.11
Health care			0.003
Unified Health System	59 (35.3)	12 (13.9)	
Health Insurance/private	108 (64.7)	74 (86.1)	
Body mass index (kg/m^2^)	26.3 ± 5.4	25.7 ± 4.0	0.40
Reason for ICU admission			0.11
Clinical	104 (62.6)	65 (75.6)	
Elective surgery	44 (26.5)	14 (16.3)	
Emergency surgery	18 (10.9)	7 (8.1)	
Charlson Index	2.8 ± 2.3	2.5 ± 1.9	0.38
Need for hospitalization in the last 12 months	80 (31.6)	47 (18.5)	0.30
Need for caregiver in the last 3 months	37 (22.2)	47 (54.7)	< 0001

ICU - intensive care unit. The results are expressed by numbers (%) or means ± standard deviations; statistical significance p ≤ 0.05; Chi square for categorical variables; analysis of variance for continuous variables.

**Table 2:** Interventions, outcomes during intensive care unit admission and muscle strength immediately after discharge

Variables	Between 61 and 79 years old (N = 167)	≥ 80 years old (N = 86)	p value
APACHE II	14.0 ± 6.8	14.3 ± 5.23	0.71
Length of ICU stay (days)	9.9 (10.4)	8.8 (8.10)	0.35
Length of hospital stay (days)	37.7 (34.3)	36.6(49.3)	0.84
Diagnosis of infection at ICU admission			
Sepsis	43 (25.9)	17 (19.8)	0.27
Septic shock	24 (14.5)	18 (20.9)	0.19
Need for vital support			
Invasive mechanical ventilation	76 (45.8)	28 (32.6)	0.04
Time of invasive mechanical ventilation (days)	8.0 ± 11.2	6.5 ± 7.6	0.51
Non-invasive mechanical ventilation	29 (17.5)	27 (31.4)	0.01
Tracheostomy	14 (8.4)	4 (4.7)	0.27
Use of vasopressor	75 (45.2)	44 (51.2)	0.37
Transfusion of blood products (red blood cells)	33 (19.9)	16 (18.6)	0.81
Transfusion of blood products (plasma or platelets)	12 (7.2)	2 (2.3)	0.11
Continuous sedoanalgesia	66 (39.8)	35 (40.7)	0.89
Conventional dialysis therapy	27 (16.3)	12 (14.0)	0.63
Continuous dialysis therapy	13 (7.8)	3 (3.5)	0.18
Outcomes during ICU			
Acute myocardial infarction	2 (1.2)	5 (5.8)	0.04
Cardiorespiratory arrest	2 (1.2)	0 (0.0)	0.31
Stroke	5 (3.1)	2 (2.3)	0.75
Acquired weakness	16 (9.6)	5 (5.8)	0.29
ARDS	6 (3.6)	1 (1.2)	0.26
Decubitus ulcer	16 (9.6)	6 (7.0)	0.48
Delirium	48 (28.9)	28 (32.6)	0.55
Nosocomial infection (pneumonia. urinary and catheter)	30 (18.1)	11 (12.8)	0.28
Conventional or continuous dialysis therapy	33 (19.8)	13 (15.1)	0.35
Muscle strength after discharge from ICU			
MRC (n = 146)	49.8 ± 9.5	47.9 ± 7.7	0.25
Dominant limb dynamometry (n = 94)	19.9 ± 9.6	14.5 ± 7.7	0.008
Non-dominant limb dynamometry (n = 93)	17.5 ± 9.1	13.1 ± 6.7	0.02

APACHE II - Acute Physiology and Chronic Health Evaluation II; ICU - intensive care unit; ARDS - acute respiratory distress syndrome; MRC - Medical Research Council. The results are expressed as numbers (%) or means ± standard deviations. Statistical significance p ≤ 0.05; Chi square for categorical variables; analysis of variance for continuous variables.

The data presented in table 3 are related to the comparison of the cumulative mortality between the ages at each follow-up point. The older elderlies had similar mortality in the third month after discharge from the ICU (26.4% *versus* 18.2, p = 0.14), and in the sixth month (26.7% *versus* 22.4%; p = 0.44) (Table 3). The older elderlies had a higher need for caregivers than the elderlies in both the third (70.9% *versus* 57.4%, p = 0.03) and sixth months (69.0% *versus* 49.5%, p = 0.002) after discharge (Table 4).

**Table 3:** Cumulative mortality over 6 months after discharge from the intensive care unit

Variables	Immediate ICU discharge N = 253			After 3 months N = 219			After 6 months N = 174		
	Between 61 and 79 years old	≥ 80 years old	p value	Between 61 and 79 years old	≥ 80 years old	p value	Between 61 and 79 years old	≥ 80 years old	p value
Deaths (%)	21 (12.9)	13 (15.1)	0.69	30 (18.2)	23 (26.4)	0.14	37(22.4)	23(26.7)	0.44

ICU - intensive care unit. Statistical significance p ≤ 0.05; Chi square for categorical variables.

**Table 4:** Consequences after discharge from intensive care unit, need for hospital care and deaths over 6 months

Variables	After 3 months N = 219			After 6 months N = 174		
	Between 61 and 79 years old	≥ 80 years old	p value	Between 61 and 79 years old	≥ 80 years old	p value
Hospitalized at the time of the interview	25/119 (21.0)	10/35 (18.2)	0.67	18/97 (18.6)	13/50 (26.0)	0.29
Need for adaptations at home	31 (25.8)	18 (32.7)	0.35	-	-	-
Need for caregiver	70/122 (57.4)	39/55 (70.9)	0.03	49/99 (49.5)	34/50 (69.0)	0.002
Number of returns to the emergency	38/100 (31.5)	18/55 (32.7)	0.39	53/128 (41.4)	27/59 (65.5)	0.31
Need for hospital readmission	27/122 (22.1)	10/55 (18.1)	0.55	41/128 (32.0)	17/59 (28.1)	0.66

ICU - intensive care unit. Statistical significance p ≤ 0.05; Chi-square for categorical variables; analysis of variance for continuous variables. There is a difference in the categories among n since not all data were obtained from all patients. The results are expressed as numbers/total (%) or only numbers (%).

**Figure 2:**
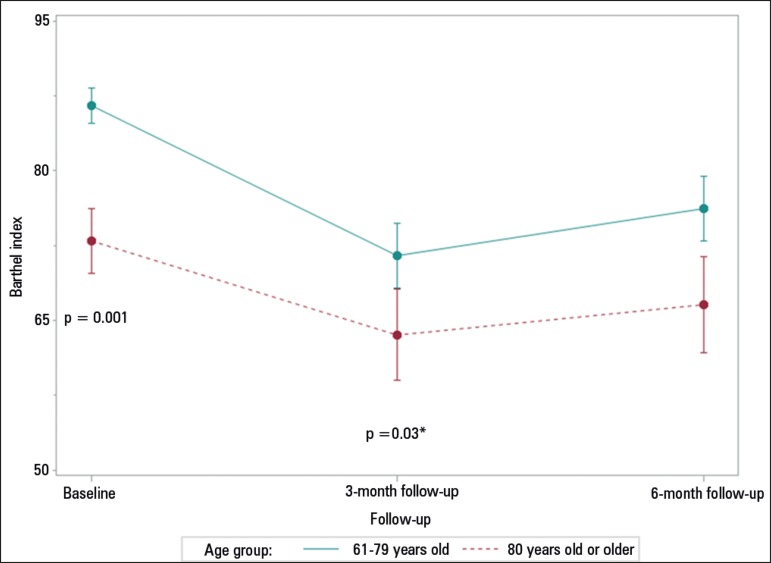
Functional evaluation (Barthel index) over six months. * Values of significance between the younger elderlies (61-69 years old) and older elderlies (80 years old or older) at each time period. Statistical significance p ≤ 0.05; Chi-square test.

Figure 2 shows that the functionality of the elderlies was worse in the older elderlies than in the younger ones prior to ICU admission and 3 months after discharge from the ICU (Barthel prior to ICU: 73.0 ± 30.0 *versus* 86.5 ± 22.6; p < 0.001; Barthel in the third month: 63.5 ± 34.0 *versus* 71.5 ± 35.5, p = 0.03), with no difference in the results of the sixth-month evaluation (p = 0.44) (Table 5). Compared to the younger elderly patients, the older elderly patients exhibited lower physical activity in the third month (in the IPAQ scoring: active/very active 3.4% *versus* 18.3%, irregularly active 32.2% *versus* 42.0%, no physical activity 64.4% *versus* 39.7%; p < 0.001) and in the sixth month (in the IPAQ scoring: active/very active 5.8% *versus* 20.8%; irregularly active 25.0% *versus* 35.9%; no physical activity 69.2% *versus* 43.4%, p = 0.005) (Table 5).

**Table 5:** Functional evaluation (Barthel index), quality of life (12-Item Short-Form Health Survey Version 2) and level of physical activity (IPAQ)

Variables	Pre-ICU			3 months			6 months		
	Between 61 and 79 years old	≥ 80 years old	p value	Between 61 and 79 years old	≥ 80 years old	p value	Between 61 and 79 years old	≥ 80 years old	p value
Barthel index (n = 253)	86.5 ± 22.6	73.0 ± 30.0	0.001	71.5 ± 35.5	63.5 ± 34.0	0.03	76.2 ± 32.3	66.6 ± 33.3	0.07
Levels of functionality									
Total dependence	7 (4.2)	7 (8.1)		22 (18.2)	12 (21.8)		16 (16.0)	9 (18.8)	
Almost total dependence	11 (6.6)	13 (15.1)		7 (5.8)	6 (10.9)		4 (4.0)	6 (12.5)	
Moderate dependence	13 (7.8)	15 (17.4)		13 (10.7)	9 (16.4)		7 (7.0)	5 (10.4)	
Little dependence	54 (32.3)	33 (38.4)		44 (36.4)	23 (41.8)		45 (45.0)	23 (47.9)	
Functional independence	82 (49.1)	18 (20.9)		35 (28.9)	5 (9.1)		28 (38.0)	5 (10.4)	
SF12v2 (n = 94)									
Physical component	-	-	-	38.0 ± 10.8	42.7 ± 8.05	0.07	40.7 ± 8.5	41.21 ± 8.3	0.85
Mental component	-	-	-	50.6 ± 11.6	47.5 ± 10.9	0.29	51.4 ± 10.8	47.55 ± 8.9	0.22
IPAQ	-	-	-			< 0.001			0.005
Active/Very active	-	-	-	24 (18.3)	2 (3.4)		22 (20.8)	3 (5.8)	
Irregularly active	-	-	-	55 (42.0)	19 (32.2)		38 (35.9)	13 (25.0)	
No physical activity	-	-	-	52 (39.7)	38 (64.4)		46 (43.4)	36 (69.2)	

ICU - intensive care unit; SF12v2 - 12-Item Short-Form Health Survey Version 2; IPAQ - International Physical Activity Questionnaire. The results are expressed as numbers (%) or means ± standard deviations; Statistical significance p ≤ 0.05; Chi-square for categorical variables; analysis of variance for continuous variables.

**Figure 3:**
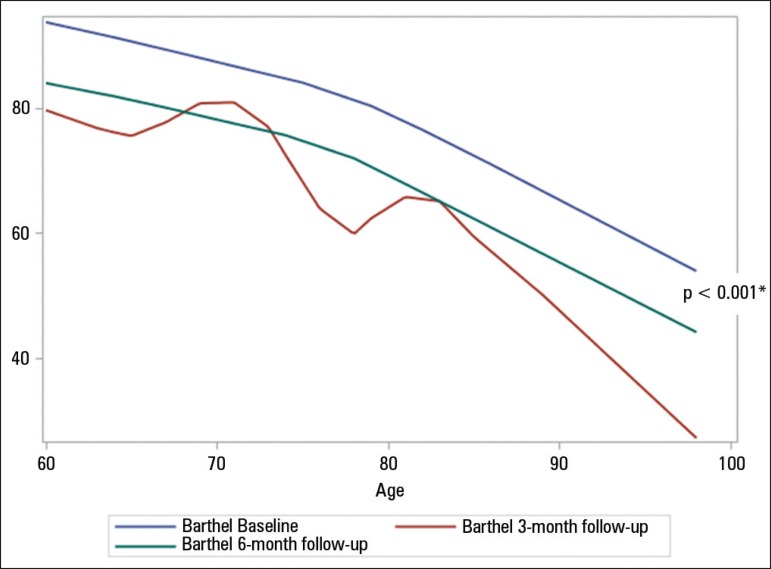
Functional design of the Barthel index in the elderlies in the follow-up at baseline, 3 months and 6 months after discharge from the intensive care unit. Significance of the functional decline over time compared with prior admission and after 6 months. Statistical significance p ≤ 0.05; analysis of variance for continuous variables.

Even considering the differences between the age groups, the loss of functionality did not differ between groups. Figure 3 shows the relationship between age and functional capacity loss, indicating that the third month assessment was not able to detect this linearity; after the sixth month, we verified that the curve was parallel when compared with the data prior to admission (p = 0.001), demonstrating that loss of functional capacity increased with age.

## DISCUSSION

Our study did not show a difference in the loss of functional capacity between younger (60 to 79 years old) and older elderlies (≥ 80 years old) in the first 6 months after discharge from the ICU; however, all presented great losses in functional capacity relative to their situation prior to hospitalization. We verified that both groups, despite their age difference, presented similar characteristics regarding the interventions during ICU admission and outcomes after immediate discharge, showing similar declines between groups over time. The same is seen in relation to comorbidities prior to ICU admission, which agrees with a previous study on predictive factors for ICU admission, which noted that chronological age alone should not be a relevant criterion to define non-admission to the ICU.^(10)^ In our sample, age groups also did not differ in terms of interventions and outcomes during ICU admission, except for the more frequent use of ventilatory support in the older elderlies group. A previous study showed a drastic decrease in the use of mechanical ventilation with increasing age.^(10)^

In our analysis, the older elderlies showed a greater functional decline in 3 months and a lower level of physical activity in 6 months, which led to a greater need for caregivers in this group. Data from previous studies have shown that elderlies benefit from aggressive interventions, but older elderlies are more likely to develop permanent disability and organ dysfunction and not to recover their baseline functional level. Furthermore, they may require long-term institutionalization and face a higher mortality risk in 5 years.^(11)^ In our study, we verified that this situation occurred in both age groups, with no difference between the two age groups.

The impact of functional status prior to ICU admission on the functional outcome after ICU discharge is of great relevance. A study that evaluated two age groups of elderly patients (65-74 years old *versus* 75 years old or older) demonstrated, as in our study, that the older elderlies had lower functionality (Katz index) in the evaluation prior to ICU admission and found no significant difference in the functionality levels of the two age groups over 1 year.^(12)^ Another study that also evaluated the functionality levels in different age groups - younger and older elderlies - did not find any differences between the two groups in ICU scores, comorbidities, length of stay in the ICU or in the main diagnoses upon ICU admission,^(13)^ similar to our study. In this study, the elderlies 75 years old or older experienced their maximum functional recovery in the 3 to 6 months after discharge, without additional improvement in one year. Autonomy in Instrumental Activities of Daily Living (IADLs) and ADLs were equal in both groups at the end of follow-up.^(13)^ After 6 months of follow-up, more than half of our patients were fully dependent regarding functionality and ability to perform ADLs. Previous studies^(12-16)^ demonstrated that patients without functional impairment in ADLs prior to ICU admission presented functional decline after critical illness compared with community controls. Only 25% of these patients recovered baseline functional levels after 1 year.^(12-14)^

With regard to dynamometry, the reference values for the elderly population are available separately for males and females and for dominant and non-dominant limbs.^(17)^ Our results did not differ by gender but showed that younger and older elderlies presented results with values below the reference levels. The differences between the two age groups were significant in both the dominant and non-dominant limbs.

Quality of life encompasses not only health status (i.e., good functional status) but also psychological factors and social and economic support. Before admission to the hospital, older elderlies had good health-related quality of life, which correlates adequately with their functional status.^(14)^ The meaning of quality of life may be different for older individuals than for younger individuals. After facing severe illness, older patients are likely to assign higher scores to their quality of life.^(14,15,17)^ A study with elderly individuals aged 80 years or older also showed that quality of life was preserved in the majority of patients after ICU admission.^(18)^

Patients in our study did not recover their functionality within 6 months compared with their pre-hospitalization levels. A multicenter Canadian cohort study, with great relevance for older elderly patients, had its sample hospitalized for an average of 7 days. After 1 year, 50% of them died, and survivors presented reduced physical function, according to the Medical Outcomes Study 36-Item Short-Form Health Survey (SF-36), compared with community controls. In our study, patients were in the ICU for an average of 9 days, and in only 6 months, 36.6% had died. In the study, only 26% of patients recovered or almost reached their prehospital level of physical functioning after 1 year.^(16)^ Old age (≥ 80 years) represents only a minor risk factor for early mortality. The most relevant factors that have the most impact on mortality at 6 months, 1 year or more after ICU admission are the number and type of comorbidities, functional status and quality of life before or shortly after ICU admission.^(15,16,19-22)^ Another study showed that the chronic conditions of the elderlies tended to be more pronounced and often occurred simultaneously at this stage. These conditions are generally not fatal but tend to significantly impair quality of life and stimulate the disabling process, a fact that may have contributed to higher mortality after discharge from the ICU. Therefore, these patients require greater care after discharge from the ICU.^(23)^

Among the strengths of our study, we emphasize that it was a prospective 6-month cohort with evaluations of elderlies and older elderlies with similar decline of groups over time and who had hospitalization of more critical patients (hospitalized for more than 72 hours, discarding elective surgeries without complications). Detailed evaluations of their comorbidities, interventions in the ICU and main outcomes after immediate and late discharge were performed. We evaluated their functional capacity using more than one method (Barthel index, MRC, upper limbs dynamometry, SF12v2 and IPAQ).

In addition to the limitations mentioned in the methods, this study was limited by the percentage of patients lost to follow up (9.8%) due to lack of telephone contact and patient information. We specifically evaluated the elderly population but did not compare it with the adult population under the same conditions. In this sense, some patients, mainly those in the older elderly group, did not present the physical or cognitive capacity to respond to the self-reported questions of the health-related quality of life and physical activity level questionnaires. However, this occurrence does not invalidate the results; by contrast, it reinforces how much age is related to dependence. Thus, the self-reported results represent elderlies with a lower level of dependence.

## CONCLUSION

There was a great loss of the functional capacity among elderly patients who stayed in the intensive care unit over 6 months when compared with the period prior to their hospitalization, with no differences between the groups of elderly and older elders.

Our results indicated that approximately half of the elderly patients admitted to the intensive care unit became functionally dependent. This finding makes us reflect on the need to rethink the admission of the elderlies to the intensive care unit, given the considerable chance they have of becoming individuals who are dependent on others but who often do not have many close relationships. This situation, however, must be analyzed on a case-by-case basis, especially in end-of-life situations, since a home-based treatment with family members might be more convenient than hospitalization in an intensive care unit, which would prolong life but without the desired quality of life.
